# Fundus flavimaculatus-like in myotonic dystrophy: a case report

**DOI:** 10.1186/s12886-021-02002-5

**Published:** 2021-05-29

**Authors:** Eric Kirkegaard-Biosca, Mònica Berges-Marti, Brahim Azarfane, Esther Cilveti, Laura Distefano, Jose García-Arumí

**Affiliations:** 1 Ophthalmology Department, Vall d’Hebron Hospital, Barcelona, Spain; 2 Ophthalmology Department, Moises Broggi Hospital, Barcelona, Spain; 3 Department of Retina and Vitreous, Instituto de Microcirugía Ocular (IMO), Barcelona, Spain

**Keywords:** Case report, Myotonic dystrophy, Pattern dystrophy, Fundus flavimaculatus

## Abstract

**Background:**

Myotonic dystrophy is an inherited disease characterized by progressive muscle weakness and myotonia. It is a multisystemic disorder that affects different parts of the body, including the eye. Dysfunction of ocular muscles, ptosis and cataract are the most common ophthalmologic manifestations, but it can also present with pigmentary changes in the retina. This report presents and discusses an unusual case of a pigmented pattern dystrophy simulating a fundus flavimaculatus in a patient with myotonic dystrophy.

**Case presentation:**

We present a case of a woman with a history of myotonic dystrophy and complaints of progressive vision loss who presented bilateral retinal pigmentary changes in posterior pole and midperiphery. The characteristics and distribution of pigmented deposits, as well as ancillary tests, showed a retinal phenotype compatible with a multifocal pattern dystrophy or a fundus flavimaculatus.

**Conclusions:**

There are a few publications about retinal disorders in patients with myotonic dystrophy. When macular area is affected it tends to adopt a patterned-shape defined as butterfly dystrophy or reticular dystrophy. To our knowledge, this is the first report of a patient with myotonic dystrophy and multifocal pattern dystrophy or fundus flavimaculatus.

## Background

Myotonic distrophy is an autosomal-dominant inherited disorder characterized by progressive wasting and weakness of the distal muscles and myotonia. This disease is caused by the expansion of a CTG repeat at two possible loci: chromosome 19 (type 1 myotonic dystrophy) or chromosome 3 (type 2 myotonic dystrophy). It is the most common adult-onset form of muscular dystrophy [1] with an approximate prevalence of 1 per 2500 in type 1 myotonic dystrophy; in type 2 myotonic dystrophy, the exact prevalence is unknown but it is underdiagnosed in the general population [2]. Typical onset occurs in the second decade.

Myotonic dystrophy is a multisystemic disease that can affect multiple organs such as heart, gastrointestinal tract, endocrine, nervous system and the eye. Ocular complications include ptosis, weakness of the ocular muscle, cataract, progressive ophthalmoplegia, exposure keratitis, low intraocular pressure and pigmentary retinal changes [3].

Pigmentary changes in the retina may lead to progressive degenerative changes in the periphery and posterior pole. Pigmentary changes at retinal pigment epithelium (RPE) of the macula can acquire a pigmented pattern shape simulating a pattern dystrophy. Here we present an atypical case of pigmentary retinal degeneration resembling a fundus flavimaculatus in a patient with myotonic dystrophy, to date never reported in the literature.

## Case presentation

A 42-year-old woman diagnosed with myotonic dystrophy at age of 15 was referred to our department in 2013 with symptoms of bilateral blurred vision and progressive vision loss. Best-corrected visual acuity (BCVA) was 20/40 in both eyes (OU). The patient presented mild bilateral ptosis with abnormal elevator function. Ocular motility was normal. Slit lamp examination revealed a moderate punctate keratopathy and minimal posterior subcapsular opacities in both lenses. Intraocular pressures (IOP) by applanation tonometry were 10 mmHg in OU.

The patient’s father and sister also suffered from myotonic dystrophy. They both presented normal slit lamp examination, only to mention the presence of a mild posterior subcapsular cataract in both cases and a low grade exposure keratitis in the sister. Fundoscopy was normal in the father and the sister, with no retinal findings.

**Fig. 1:**
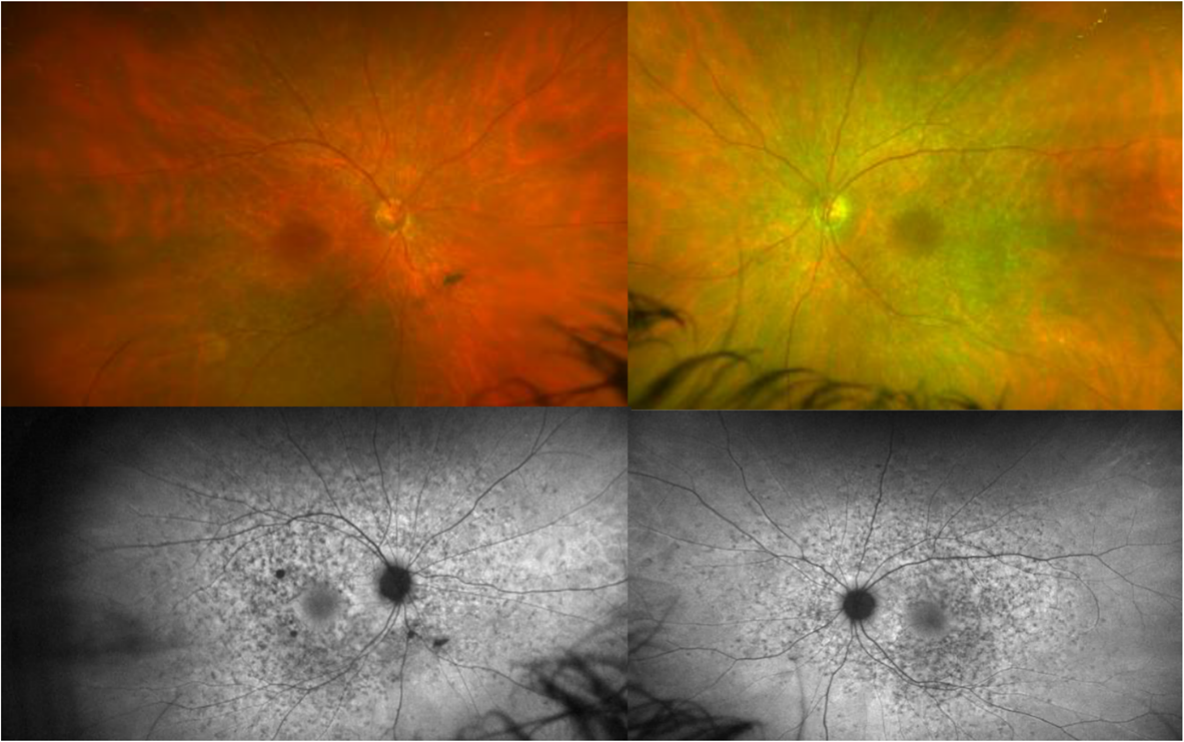
Optomap ultra-widefield funduscopy (Optos®, Optomap®, UK) shows multiple yellowish deposits throughout the posterior pole and midperiphery. Ultra-widefield fundus autofluorescence (Optos®, Optomap®, UK) reveals multiple patched areas, some of them with increased autofluorescence, in the posterior pole and midperiphery. The fovea and the peripapillary area are respected

Dilated fundus examination of the case reported showed multiple deep yellowish deposits scattered throughout the posterior pole and extending out to midperiphery, sparing the peripapillary area and with a moderate degree of macular atrophy in OU resembling a fundus flavimaculatus (Fig. 1).

Fundus autofluorescence (FAF) imaging showed multiple patched areas of increased autofluorescence probably corresponding to lipofuscin within the flecks, surrounded by regions of decreased autofluorescence from adjacent atrophic RPE in the posterior pole and midperiphery. The central 1500 μm of the fovea and the peripapillary area were respected (Fig. 1).

**Fig. 2:**
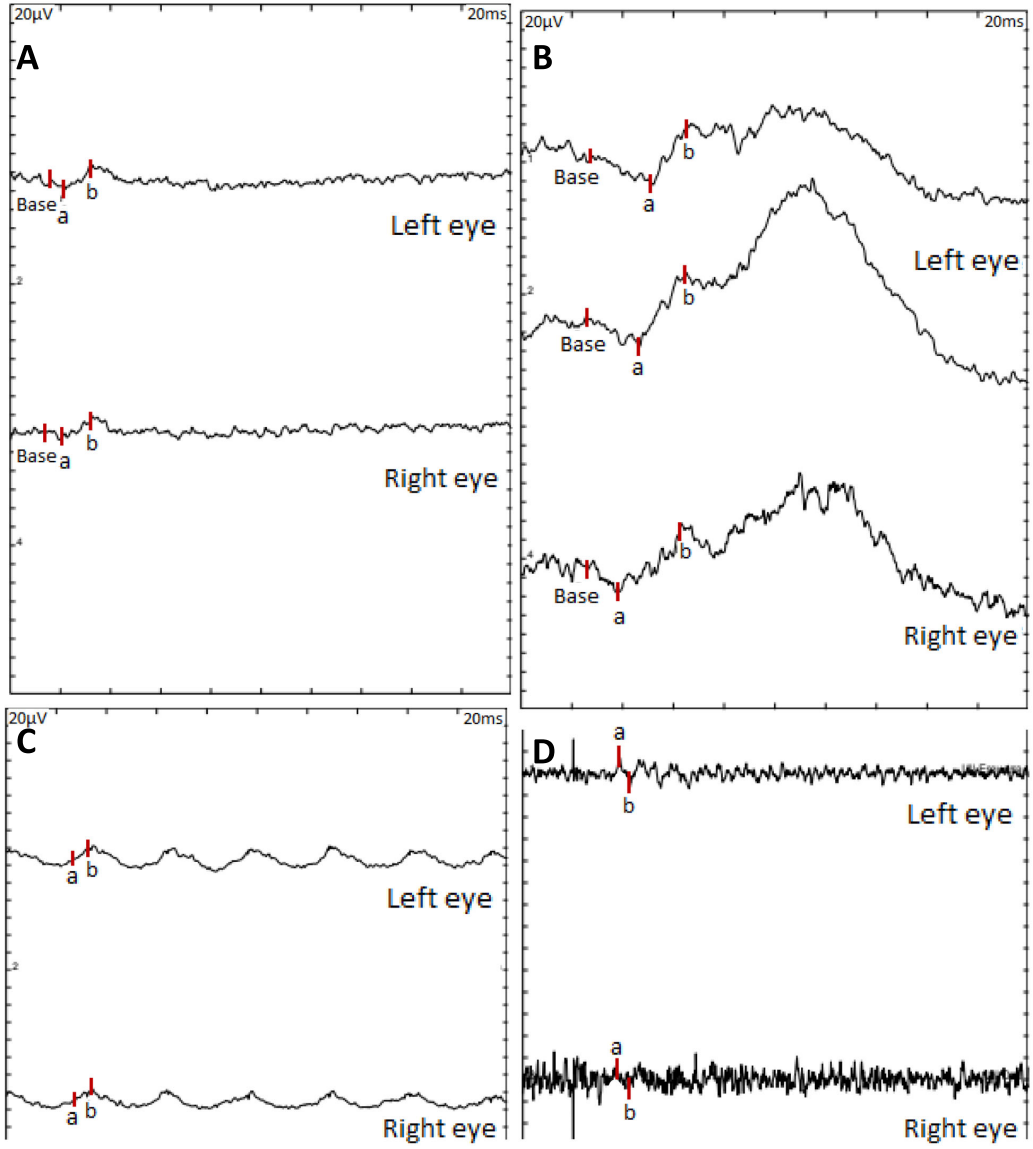
Full-field electroretinogram (ffERG). Image **A**: 3.0 ffERG under dark-adapted conditions (rod-cone response). Image **B**: 0.01 ffERG (rod response). Image **C**: 30 Hz flicker (light adaptation, cone response). Image **D**: 3.0 ffERG oscillatory potencials (amacrine cells). A decreased amplitude is obtained in OU under photopic (**A** and **C**) and scotopic (**B** and **D**) conditions, more severe in OD

Regarding electrophysiological test, the patient presented normal visual evoked potential but pathological full-field electroretinography in OU, showing reduced amplitudes in photopic and scotopic conditions more severe in right eye (OD) (Fig. 2). The electrooculography revealed an infranormal Arden ratio in OU (OD 1.64, OI 1.63), findings that were compatible with a RPE dysfunction.

**Fig. 3:**
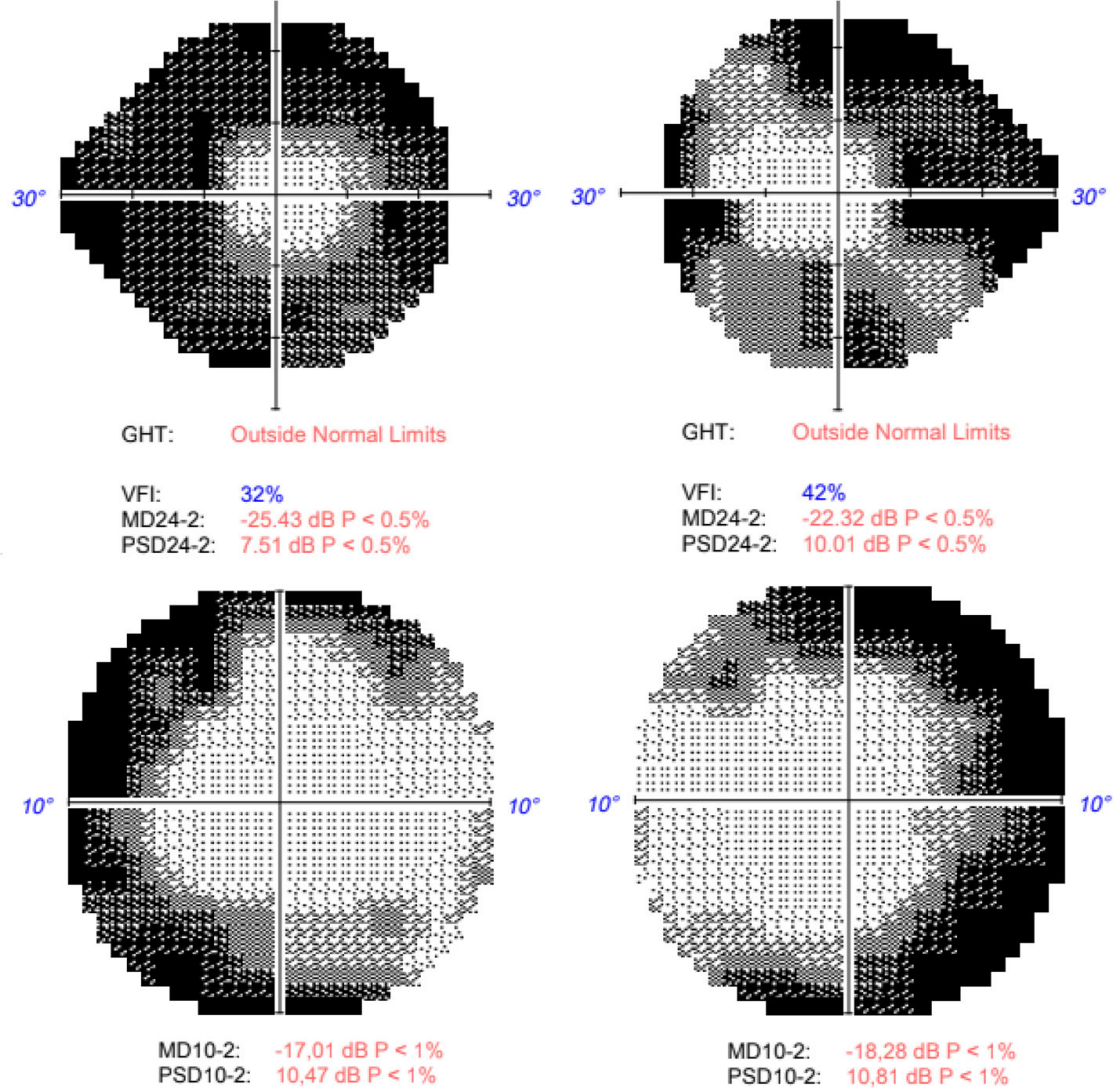
24–2 Humphrey visual field with a severe peripheral visual loss in OU. 10–2 Humphrey visual field (VF) with temporary involvement in OU

24–2 Humphrey visual field (VF) revealed a severe peripheral visual loss in OU. Subsequent annual 10–2 Humphrey VF were performed with non-progressive temporary involvement (Fig. 3).

**Fig. 4:**
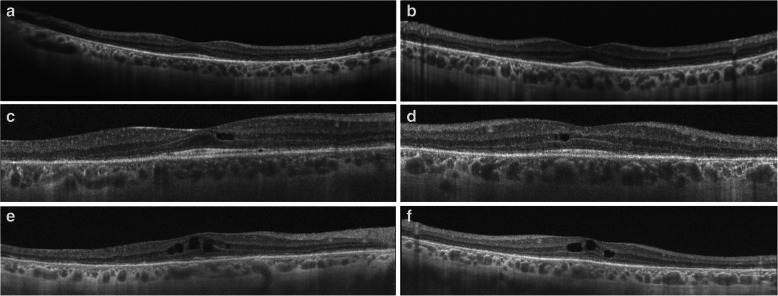
Optical coherence tomography (Swept Source OCT, Triton^TM^, TOPCON, Japan) images of the macula of OU in 2015 (**a**, **b**). Note the atrophy and loss of external layers except in the foveal area. Optical coherence tomography images of the macula of OU in 2017 (**c**, **d**) and in 2019 (**e**, **f**) show progressive appearance of hiporreflective intraretinal cavities. In **e** and **f** images it also seems to be an increased degree of atrophy in subfoveal external layers

Optical coherence tomography (OCT) images revealed atrophic RPE and retinal thinning with foveal sparing in OU. No subretinal or intraretinal fluid was observed (Fig. 4).

The patient was followed-up every 6–12 months for 7 years with no remarkable changes at slit-lamp or fundoscopy examination. IOP, measured by applanation tonometry, have always been low (between 6 mmHg and 12 mmHg depending on the visits). BCVA on the last visit performed in october 2020 was 20/60 in OD and 20/50 in left eye (OS).

No progression was observed in FAF and VF performed annually. However, subsequent OCT scans showed a slow progression of atrophy in OU over the years, also with an involvement of the inner layers and the appearance of intraretinal hyporeflective cavities in OU (Fig. 4).

## Discussion

Myotonic dystrophy is a genetic neuromuscular disease characterized by myotonia, muscle weakness and atrophy. It is a slowly progressive and multisystemic disorder that can produce cardiac, gastrointestinal, neurological and ophthalmological problems, among others. Regarding the eyes, it is typical the affectation of periocular muscles, the presence of ptosis, cataract and in some patients the appearance of pigmentary changes in the RPE that can occur in the peripheral retina or in the posterior pole. Macular pigment changes occur in 20% of patients with myotonic dystrophy [4] usually having pattern dystrophy like presentation.

Pigmented pattern dystrophies are an inherited disorder that typically manifest in the second to fifth decade of life. They tend to be bilateral, symmetric, slow progressive and therefore less devastating to visual function, with symptoms of vision loss, metamorphopsia, nyctalopia or central scotoma [5]. Most of the patients remain asymptomatic until late stages, where the atrophy of the RPE begins or choroidal neovascularization develops [6]. Pattern dystrophies are usually a group of hereditary retinal diseases mostly related to PRPH2 gene variants, usually in an autosomal dominant inheritance although they can also present in an autosomal recessive inheritance [7].

The presence of multiple irregular yellowish lesions in the posterior pole and midperiphery that respect the peripapillary area is known as fundus flavimaculatus. This phenotype is strongly related to Stargardt disease, which is the most common macular dystrophy affecting 1:8000 to 1:10000 people worldwide. In 90% of patients it is an autosomal recessive condition due to variants in ABCA4 gen [7, 8]. Variants in ELOVL4 gen and PROM1 gen have also been described in Stargardt disease in an autosomal dominant pattern [7].

Pigmented pattern dystrophies are a heterogeneous group of diseases that include different phenotypes depending on the distribution of pigment deposition above the RPE within the macular area. They can be divided in five groups according to the pattern form adopted by the distribution of these pigmented deposits [9, 10]. The first group is adult-onset foveomacular vitelliform. It initially appears as a bilateral yellowish oval deposit at the macula that tends to disappear and evolve to atrophy in late stages [11]. The second group is butterfly dystrophy. It is characterized by bilateral accumulation of pigmented or yellowish material at resembling the wings of a butterfly [12]. Like the other groups, the pigmentation initially increases, with slow depigmentation and the appearance of atrophic changes in late stages [9]. The third group is reticular dystrophy. It appears as an initial pigmented lesion within the macula that is accompanied by a network of granular pigmented deposits radial to the central point, resembling a ‘fishnet’, all limited to the posterior pole [13]. Some specific phenotypes are included in this group, such as spider dystrophy, also known as macroreticular dystrophy [14]. The fourth group is multifocal pattern dystrophy. Fundoscopically, multiple regular yellowship lesions can be found scattered in posterior pole and periphery simulating fundus flavimaculatus. Sometimes the only way to distinguish it from Stargardt disease is to complete a genetic study or to perform a fluorescein angiography, where the characteristic choroidal silence of Stargardt disease would not be observed [15]. The fifth group is fundus pulvurentus. Presentation includes a pigment mottling in the macular area resembling a salt and pepper appearance which can spread to midperiphery [5, 10]. This classification is based on fundus appearance. However, there may be mixed or incomplete forms which makes it difficult to distinguish a specific group, so they are usually defined as pattern dystrophy in general.

The case presented could correspond to the fourth group of pattern dystrophies (multifocal pattern dystrophy), which is indistinguishable from fundus flavimaculatus. Cases of pattern dystrophy in patients with myotonic dystrophy is an uncommon but documented phenomenon. Kimizuka et al. analyzed a series of 49 patients (98 eyes) in patients with myotonic dystrophy and they found in 26 eyes (26.6%) butterfly-shapped macular pigmentary changes, in 24 eyes (24.5%) reticular pigmentary changes and in 43 eyes (43,9%) peripheral atrophic polygonal-shaped changes [16]. There are a couple of studies in the Korean population that analyze ophthalmological findings in these types of patients [17, 18], with a reported incidence of pigmentary changes in the retina much lower than Kimizuka et al. There are also a few reported cases of patients with myotonic dystrophy and retinal butterfly dystrophy [19, 20] and spider dystrophy [14] but, to our knowledge, there are no other reported cases that associate myotonic dystrophy with other retinal dystrophies.

Unfortunately, no genetic study has been performed during the follow-up of the patient. It would be interesting to complete the study in search of the most common genetic causes of this phenotype (PRPH2, ABCA4) in order to be able to confirm the diagnosis and the subtype of dystrophy.

## Conclusion

Myotonic dystrophy is a disorder that produces progressive wasting, weakness and myotonia of distal muscles. It affects multiples systems, including the eye, typically producing ptosis, weakness of periocular muscles, cataract and also retinal pigmentary changes in periphery and posterior pole. When pigment deposits are located within the macular area, they usually adopt a patterned-shape that can be classified as a pigmented pattern dystrophy. In our case there is no genetic study done so we cannot establish a direct relation between the mutation causing myotonic dystrophy and retinal dystrophy, but there are some reported cases of association between myotonic dystrophy with butterfly-patterned dystrophy and reticular-patterned dystrophy. It seems logical to think that a relationship between these two diseases could exist. To our knowledge, this would be the first report of a patient with myotonic dystrophy and fundus flavimaculatus or multifocal pattern dystrophy.

## Data Availability

All data generated or analysed during this study are included in this published article.

## Abbreviations

BCVA: Best-corrected visual acuity
FAF: Fundus autofluorescence
ffERG: Full-field electroretinogram
IOP: Intraocular pressure
OCT: Optical coherence tomography
OD: Right eye
OS: Left eye
OU: Both eyes
RPE: Retinal pigment epithelium
VF: Visual field
